# Congenital Abdominal Aortic Aneurysm: A Case Report and Literature Review

**DOI:** 10.3389/fped.2022.853517

**Published:** 2022-03-01

**Authors:** Zhibin Zhou, Yongqiang Yue, Ke Ma, Zhaohui Hua, Zhen Li

**Affiliations:** Department of Endovascular Surgery, The First Affiliated Hospital of Zhengzhou University, Zhengzhou, China

**Keywords:** aortic aneurysm, aortic diseases, congenital, children, surgical treatment

## Abstract

Congenital abdominal aortic aneurysm is a rare disease with unknown etiology, and the common symptoms are abdominal pulsatile mass and pain caused by aneurysm rupture. The disease has a high mortality rate and fewer reports of surgical treatment. Here, we present a case of an idiopathic congenital abdominal aortic aneurysm. A 4-year-old boy had an abdominal pulsatile mass, and computed tomography angiography revealed an isolated infrarenal abdominal aortic aneurysm. To prevent rupture of the aneurysm, we repaired the aneurysm with artificial graft transplantation. No genetic mutation of the known congenital aneurysmal diseases was found in the whole-exome sequencing of the patient and his parents. There was no graft obstruction, and the patient grew well 40 months after surgery. Open surgery is the best treatment for idiopathic congenital abdominal aortic aneurysms. Surgical details such as timing and graft selection need to be further explored.

## Introduction

Abdominal aortic aneurysms in children are very rare. The common causes are congenital connective tissue disorders, vasculitis, traumatic umbilical artery intubation, and infection, while congenital abdominal aortic aneurysms (cAAAs) is rarely reported and has an unknown etiology and high mortality. This article reports the case of a 4-year-old child with isolated cAAA and the results of a 40-month follow-up of open surgery.

## Case Description

**Figure 1:**
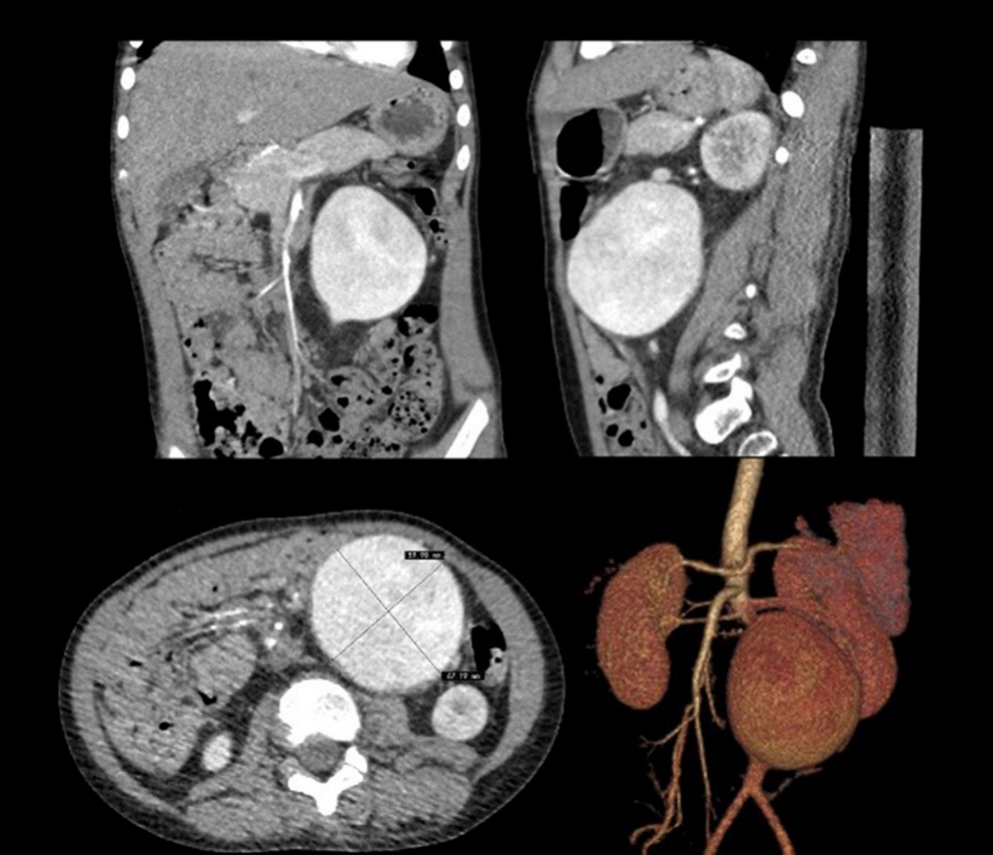
Computerized tomography angiography demonstrated isolated infrarenal abdominal aortic aneurysm.

In June 2018, a 4-year-old boy was hospitalized because of the discovery of a left abdominal pulsatile mass for 2 months. Computed tomography angiography (CTA) revealed an isolated infrarenal AAA with a maximum diameter of 67 mm (Figure 1). The patient was 112 cm tall and weighs 23 kg and he had no family history of aneurysmal disease, connective tissue disorders, a history of trauma, umbilical cannulation, and infection. Laboratory tests revealed no abnormalities, and blood pressure was normal.

## Diagnostic Assessment, Therapeutic Intervention, Follow-Up, and Outcome

**Figure 2:**
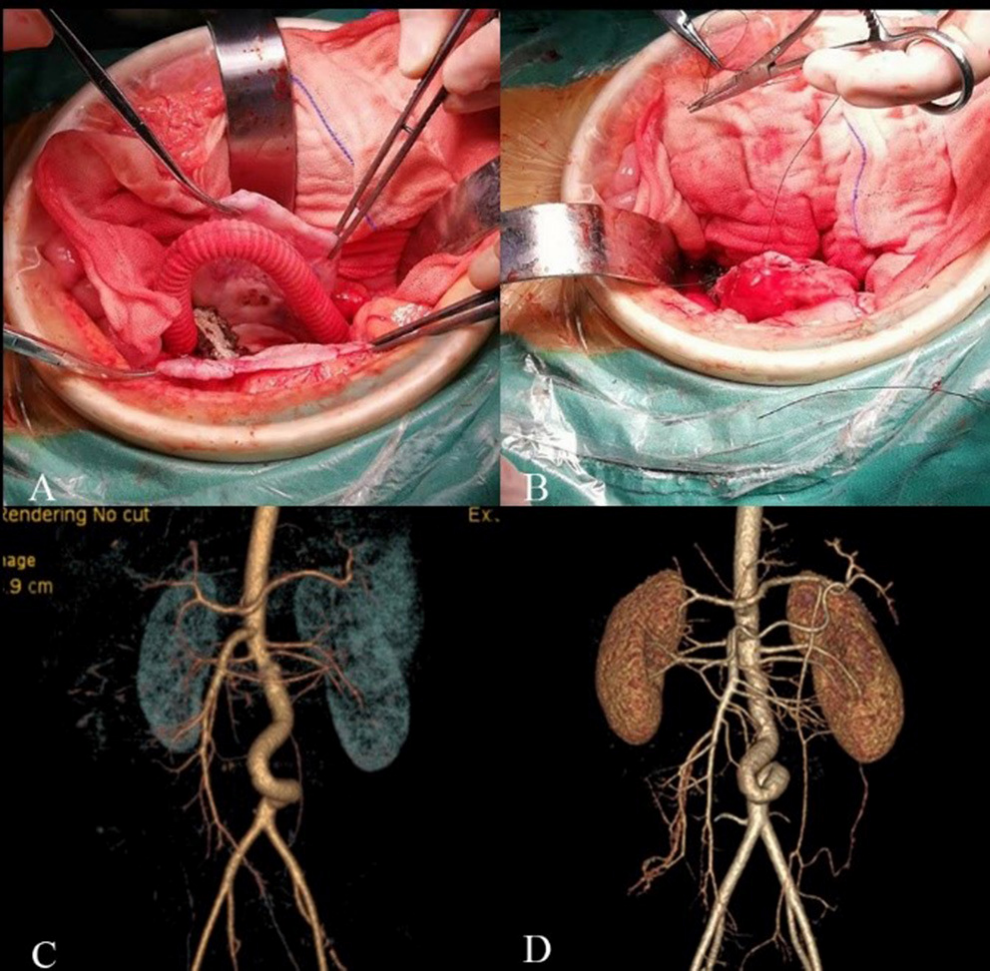
Intraoperative and follow-up images. **(A)** A 10 mm Dacron aorto-aortic tube graft replace the AAA with a “C” shape. **(B)** The aneurysmal sac was wrapped around the graft. **(C)** CTA 7 days after surgery. **(D)** CTA 40 months after surgery showed twisted but unobstructed graft.

In July 2018, we repaired the AAA using open surgery to prevent aneurysm rupture. When we blocked the blood flow at both ends of the aneurysm and opened the aneurysm through a midline incision, we found that the intima of the aneurysm was smooth without thrombus. We then used a 10-mm Dacron aorto-aortic tube graft to replace the AAA; the graft was oversized by 6 cm and formed a “C” shape to allow aortic growth. The aneurysmal sac was wrapped around the graft to avoid aortoduodenal fistula. The patient recovered well after the operation. Whole-exome sequencing of the boy and his parents revealed no genetic mutations of the known congenital aneurysmal diseases. The patients had frequent follow-ups outside the hospital, and at 40 months post-operative follow up without any antiplatelet drugs, the patient was 140 cm tall and weighs 43 kg, and the CTA revealed that the graft blood flow was unobstructed (Figure 2).

## Discussion

AAA is more common in the elderly with arteriosclerosis, and is rare in children and infants, and is commonly caused by congenital connective tissue disorders, vasculitis, umbilical cannulation, and infection, while cAAA is extremely rare and has an unknown etiology. One relevant hypothesis is that cAAA results from a developmental defect during embryogenesis that creates a focal narrowing of the abdominal aorta, which leads to poststenotic turbulent blood flow and subsequent aneurysm formation (1). There are no epidemiological data related to cAAA, and as of December 2021, only 31 cases have been reported (Table 1) (2–8). There are fifteen patients diagnosed before 1 year of age, eight patients were diagnosed after 1 year of age, and the remaining eight patients were diagnosed at 19–30 weeks of gestation. The male-to-female ratio was 18:10, 20 of 31 patients had infrarenal AAA, and 19 of 30 patients had other aneurysms. The reason for admission of patients is usually abdominal pulsatile mass or rupture of aneurysm; suspected diseases should receive vital imaging examination, such as ultrasound, which can provide a clear diagnosis.

**Table 1:** Previously reported cases of idiopathic congenital abdominal aortic aneurysms.

**Author**	**Gender**	**Age at discovery**	**Location**	**Other aneurysms**	**Other aneurysms**	**Surgical treatment**	**Outcome**
Howorth Jr. MB	Female	1 day	Infrarenal	None	Large abdominal mass, vomiting, anorexia	Exploratory laparotomy	Ruputure and death during operation
Darden WA	Male	2.5 years	Infrarenal	None	None	Dacron aortic graft	Died of pneumonitis at 5 months after surgery
Sterpetti AV	Male	19 years	Infrarenal	None	Middle epigastric pain, abdominal fullness, dysuria, abdominal pulsatile mass	Dacron aortic graft 18 mm	Died of pneumonitis at 5 months after surgery
Odagiri S	Male	1 year	Infrarenal	Multiple left renal artery aneurysrre, bilateral corrmon iliac artery aneurysms	None	Dacron aortic graft 12 mm	Healthy at 10 months after surgery
Latter D	Male	1 month	Infrarenal	None	Pulsatile abdominal mass	Polytetrafluoroethylene tube graft 8 mm	Healthy at 10 months after surgery
Saad SA	Male	6 weeks	Infrarenal	Left common iliac artery aneurysm mass	Pulsatile abdominal mass	Aneurysmorrhaphy	Healthy at 3 months after surgery
Myrmel T	Male	30 years	Infrarenal	None	Pulsatile abdominal mass, acute abdominal pain	Albumin coated USCI graft sized 16 × 8 mm	Healthy at 1 year after surgery
Malee MP	Female	32 weeks' gestation	Juxtarenal	Aneurysmal dilation of the bilateral iliac artery (details unknown)	Palpable abdominal mass, ileus compression from an aneursysm	None	Died of acute pulmonary hypertension and cardiac dysfuncticn at age 9 days
Kim ES	Female	9 days	Juxtarenal	None	None	None	Died of heart failure secondary to renovascular hypertension at age 20 days
Mehall JR	Male	6 weeks	Juxtarenal	Right common iliac artery aneurysm	None	Bifurcated GoreTex graft 7–4 mm	Healthy at 1 month after surgery
Laing AJ	Male	12 months	Infrarenal	None	Pale, shocked, in an urresponsive state, vomiting, abdominal distention	Exploratory laparotomy	Rupture and death during operation
Dittrick K	Male	12 years	Infrarenal	None	None	Collagen impregnated Dacron aortic graft 14 mm	Healthy at 2 years after surgery
Bell P	Female	1 day	Infrarenal	None	Billous vomiting, large abdominal mass	Cryopreserved allograft 5 mm	Healthy at 14 months after surgery
Cheung SCW	Male	6 months	Juxtarenal	Bilateral common and external iliac artery aneurysms, right internal iliac artery aneurysm	None	None	Progression of thrombosis of the aneurysm and renal dysfunction at age 3 years
Buddingh KT	Male	1 day	Juxtarenal	Descending thoracic aortic aneurysm, left common iliac artery aneurysm	Bilous vomiting, anorexia, pulsatile abdominal mass	None	Alive at 7 months, aneurysm has grown to a maximum diameter of 93 mm
Kim JI	None reported	21 weeks' gestation	Infrarenal	Bilateral common iliac artery aneurysms, left internal iliac artery aneurysm	None	Dacron aortic graft 12 mm	Uneventful postoperative recovery
Malikov S	Male	28 weeks' gestation	Juxtarenal	None	Pulsatile abdominal mass	Repair with native iliac vessels	Healthy at 39 months after surgery
Cantinotti M	None reported	22 weeks' gestation	Unspecified	None reported	None reported	None reported	None reported
Tsunematsu R	Male	25 weeks' gestation	Unspecified	None	Pulsatile abdominal mass	None	Stable after 6 months follow up
McAteer J	Female	32 weeks' gestation	Thoracoab dominal	None	None	None	Died of rupture at age 4 weeks
Cho YP	Male	23 months	Infrarenal	None	Irritability, vomiting, poor oral intake, diffuse tenderness, palpable pulsatile abdominal mass	Cryopreserved cadaveric artery 7 mm	Healthy at 10 months after surgery
Meyers RL	None reported	Neonate	Infrarenal	None	None	Decellularised, antigen reduced cryopreserved allograft	Healthy at 29 months after surgery
Ko Y	Male	2 months	Supraceliac	Two descending thoracic aortic aneurysms	None reported	Dacron aortic graft 10 mm	Uneventful postoperative recovery
Fettah ND	Female	1 day	Infrarenal	None	Vomiting, abdominal distention, palpable pulsatile abdominal mass	Repair with polytetrafluorethylene patch	Died of sepsis and cardiopulmonary insufficiency at 4 weeks after surgery
Bivins HS	Male	19 weeks' gestation	Infrarenal	Iliac artery aneurysms (details unknown)	Large abdominal mass	None	Died of renal failure at age 12 days
Bansal A	Male	1 year	Infrarenal	None	Abdominal distension	Dacron aortic graft 10 mm	Uneventful postoperative recovery
Sirisabya A	Female	1 day	Infrarenal	Left common iliac artery aneurysm, two small right renal artery aneurysms	Marked abdominal distension with a large pulsatile mass	Gore-Tex vascular graft	Thrombosis of the aortic graft and bilateral common iliac, internal iliac, and external iliac arteries at 13 months after surgery. Living a fairly normal life at 26 months after surgery
Kuboi T	Female	Neonate	Infrarenal	None	Lower back mass (subcutaneous vascular malformation)	None reported	None reported
Higuchi K	Female	4 years	Infrarenal	Multiple intracranial aneurysms, bilateral hypogastric artery aneurysms, left renal artery aneurysm	Palpable pulsatile abdominal mass	Dacron aortic graft 10 mm	Healthy at 21 months after surgery
Tanga C F	Male	11 years	Infrarenal	Bilateral common iliac artery aneurysms, bilateral internal iliac artery aneurysm	Abdominal pain, shock	Dacron aortic graft 12 mm	Healthy at 10 months after surgery
LeNguyen A	Female	36 weeks' gestation	Infrarenal	Bilateral common iliac artery aneurysms, bilateral internal iliac artery aneurysm	None	Cryopreserved cadaveric artery 5 mm	Healthy at 12 months after surgery

*Modified from Wang and Tao (2)*.

The histopathological changes in the intima of cAAA include calcifications, thromboses, and ulcerations and ruptures of the layers (2, 9). Molecular genetic defects considered to be associated with AAA, Marfan's syndrome, and Loeys-Dietz syndrome are caused by mutations in the genes encoding TGF-β2 or TGF-β receptor (TGFBR) I or II. Mutations in the fibrillin-1(FBN1) gene have also been found to be associated with the occurrence of coronary aneurysms (10–13). Unfortunately, no similar genetic or molecular changes have been found in cAAA, including this case.

The mortality caused by cAAA rupture and renal failure was 30.76% (2). There is no universal approach to the management of cAAA. Although steroids, cyclophosphamide, antihypertensive drugs, non-steroidal anti-inflammatory drugs, and statins have certain curative effects, the reported mortality of conservative treatment is still as high as 57.14% (4/7). It is still unclear how to judge the diameter of aneurysms in the intervention, and the uncertainty of children's activity cannot refer to the surgical standards of adults. Surgical repair after diagnosis should be considered. Endovascular aneurysm repair (EVAR) is not feasible in infants or children because of the lack of an appropriate endograft and the impact on patients' growth and development. Artificial grafts and allografts were most frequently selected for revascularization, and in the past, 13 cases were reported using Dacron graft or polytetrafluoroethylene (PTFE) graft, 4 cases of allografts, and 1 case of native vessels. Although allografts have the advantages of high long-term patency and low risk of postoperative graft infection, there are difficulties with the long-term use of immune-suppressants and allograft sources. Malikov reported a successful case of revascularization with native iliac vessels (14).

The common complications of artificial vascular grafts are graft stenosis and obstruction. The diameter of the artificial graft should be considered to match with the artery and ensure blood supply to the lower limbs. At present, the reported diameter is mostly between 8 and 12 mm, which is easier for older children to choose. There is a high risk of synthetic vascular graft occlusion with a diameter of <6 mm for a neonatal patient; it may, therefore, be more appropriate to delay surgery for smaller aneurysms to produce better results and prevent the need for follow-up surgery (15). It is necessary to reserve appropriate length for artificial grafts to adapt to the patient's growth; however, most reports do not indicate the specific appropriate length. Dueppers et al. (16) reserved 4-cm graft to meet the growth needs of children. We considered the patient's age, preoperative aortic diameter and adult physique estimated from his parents' physique, and decided to use 10-mm Dacron graft to reconstruct the diseased artery with a 6-cm long graft reserved to form a “C” shape; our length selection principle is to keep a certain length on the premise of avoiding angulation.

The average follow-up time of the 16 patients who underwent surgical repair with follow-up records was 19 months. The longest follow-up time with artificial grafts under the age of 18 years was 26 months. Of these 16 patients, two died of infection during follow-up, an anastomotic stenosis of allografts occurred 7 days after surgery, and one PTFE graft was completely occluded 13 months after the operation. The graft patency rate was 93.3% (14/15). There is no literature recommending the routine use of anticoagulant or antiplatelet drugs for AAA patients with reconstructed branches. LeNguyen et al. (8) continued to use low-molecular-weight heparin for patients with anastomotic stenosis, resulting in graft patency at 1-year follow-up.

During the 40-month follow-up in our case, the artificial graft was twisted, but there was no obvious angulation and it remained unobstructed. The patient's physical development was not affected. We will continue to follow up the patient to observe graft patency.

CAAA is rare and has unknown etiology, and for patients with confirmed aneurysms, it is suggested to improve the systemic examination and long-term follow-up to exclude other lesions. Due to the high mortality rate, the long-term results of open repair in children are still unclear. The patency of grafts also requires long-term follow-up observations and necessary drug adjuvant treatment. In cases of complications, timely and effective interventions are necessary.

## Data Availability Statement

The original contributions presented in the study are included in the article/supplementary material, further inquiries can be directed to the corresponding authors.

## Ethics Statement

The studies involving human participants were reviewed and approved by Medical Ethical Committee of the First Affiliated Hospital of Zhengzhou University. Written informed consent to participate in this study was provided by the participants' legal guardian/next of kin. Written informed consent was obtained from the minor(s)' legal guardian/next of kin for the publication of any potentially identifiable images or data included in this article.

## Author Contributions

ZZ was wrote the manuscript and was assistant in surgery. KM and YY were assistant in surgery and participate in editing the articles. ZH and ZL designed the operation and revising the manuscript. All authors contributed to the article and approved the submitted version.

## Funding

This research was supported by National Natural Science Foundation of China (8187020205), and its main expenditure is layout fee.

## Conflict of Interest

The authors declare that the research was conducted in the absence of any commercial or financial relationships that could be construed as a potential conflict of interest.

## Publisher's Note

All claims expressed in this article are solely those of the authors and do not necessarily represent those of their affiliated organizations, or those of the publisher, the editors and the reviewers. Any product that may be evaluated in this article, or claim that may be made by its manufacturer, is not guaranteed or endorsed by the publisher.
